# Supplementary material for: Risk Factors Associated with 30-Day Mortality in Older Patients with Influenza
Source: J Clin Med. 2021 Aug 11;10(16):3521. doi: 10.3390/jcm10163521 (PMC8396973; doi:10.3390/jcm10163521)
Supplement: Supplementary file 1 [file jcm-10-03521-s001.zip › jcm-1317117-supplementary.pdf]

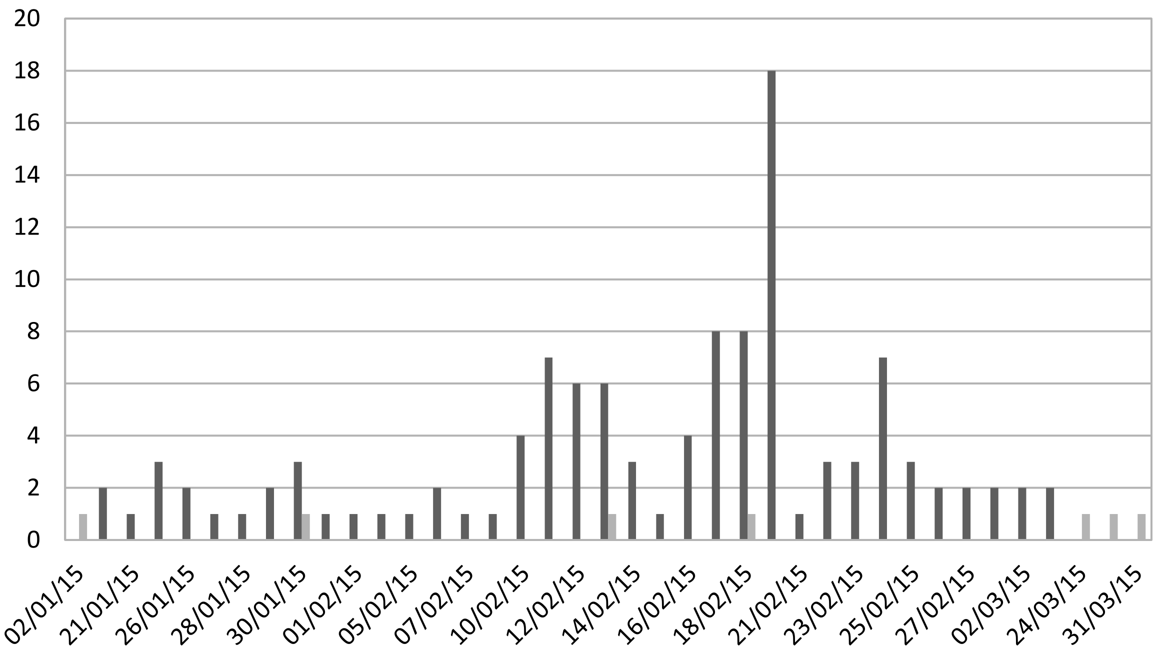

Supplementary Figure S1. Distribution over time of the number of positive influenza RT-PCR, (n=122). In black: positive RT-PCR for detection of influenza A. In grey: positive RT-PCR for detection of influenza B.

Supplementary Table S1. Screening, non-inclusion and enrollment process of participants according to the site of inclusion (Clinical Gerontology Department).

Unit	Patients Screened N=122	Patients Non-Included N = 8	Patients Included N=114
Acute-care unit n°1	42	4	38
Acute-care unit n°2	26		26
Post-acute care services n°1	7	1	6
Post-acute care services n°2	3		3
Long term care facilities	16	2	14
Nursing home residence n°1	10		10
Nursing home residence n°2	18	1	17
